# Tablet-based assessment protocol for cognition: development and preliminary usability testing of a cognitive screening tool

**DOI:** 10.3389/fpsyg.2026.1798377

**Published:** 2026-04-08

**Authors:** Denise Mellace, Roberto Prandin, Carlo Manzoni, Angelica De Sandi, Sara Marceglia, Stefano Zago, Roberta Ferrucci

**Affiliations:** 1 Department of Oncology and Hemato-Oncology, University of Milan, Milan, Italy; 2 Foundation IRCCS Ca’ Granda Ospedale Maggiore Policlinico, Milan, Italy; 3 Department of Health Sciences, University of Milan, Milan, Italy

**Keywords:** cognitive screening, early detection, neuropsychological tests, touch screen tablet, usability

## Abstract

**Introduction:**

Computerized cognitive screening offers several advantages over traditional paper-and-pencil methods, including standardized administration, automated scoring, and scalable deployment. Touchscreen-based tools provide an intuitive and accessible interface for digital screening in both clinical and non-clinical contexts. However, there is continued interest in developing brief digital home-deliverable screening tools that encompass multiple cognitive domains and in systematically addressing usability during tool development.

**Methods:**

This paper presents the development and preliminary usability evaluation of TAP-COG, a tablet-based cognitive screening tool assessing processing speed, sustained attention, inhibitory control, short-term memory, and resistance to interference. TAP-COG comprises five self-administered tasks—Visual Reaction Time, Go/No-Go, Visual Recognition, Simon, and Interference—optimized for tablet interaction, requiring approximately 30 min to complete. Key performance metrics (reaction time, accuracy, errors) are automatically recorded. We conducted a usability study with 80 healthy adults (mean ± SD age: 40.75 ± 17.72; Montreal Cognitive Assessment: 28.45 ± 1.72) using the System Usability Scale.

**Results:**

All participants completed the battery independently and reported clear instructions and ease of use. Overall, 80% rated usability as “good” to “excellent,” with no significant influence of age, education levels, or cognitive status on usability ratings.

**Conclusion:**

TAP-COG demonstrates high usability, supporting its potential as a scalable, self-administered cognitive screening tool. These findings justify further validation in larger and more diverse populations and highlight TAP-COG’s promise for both research and clinical applications.

## 1 Introduction

In recent years, neuropsychological testing has increasingly shifted from traditional paper-and-pencil assessment to computerized and digital formats. While conventional assessments remain widely used in clinical and research settings, they are often time-consuming, expensive, and limited in ecological validity (Morrison et al., 2015). These limitations have motivated the development of more accessible, standardized, and scalable digital solutions.

Although digital cognitive tools have been in development for decades (Wild et al., 2008; Bauer et al., 2012), the COVID-19 pandemic accelerated the demand for remote and flexible assessment methods and boosted the global shift toward digital health and telemedicine (Ruggiero et al., 2024). Disruptions to in-person services underscored the shortcomings of face-to-face testing and emphasized the need for tools that can be administered remotely with minimal supervision. Digital platforms also allow broader reach, enabling assessment of populations living in remote areas or facing logistical or mobility barriers in accessing healthcare facilities (Mameli et al., 2022).

One of the main factors driving the adoption of computerized testing is its growing application in cognitive impairment screening (Roebuck-Spencer et al., 2017), particularly for early detection of neurocognitive disorders (García-Casal et al., 2017). In general, structured cognitive screening facilitates the detection of cognitive impairments, helps identify individuals who require further evaluation, and provide baseline measures for longitudinal monitoring. Such tools are typically quick to administer, and require minimal staff training (Boustani et al., 2003; Cordell et al., 2013). Computerized screening tools, in particular, offer a range of operational advantages over traditional paper-based methods, such as standardized administration protocols, precise stimulus presentation, accurate response recording, and automated scoring (Morrison et al., 2015). Several digital cognitive tools have also demonstrated good reliability and construct validity across multiple domains (Moore et al., 2017; Dali et al., 2024), as well as enhanced ecological validity when implemented in real-world settings (Sliwinski et al., 2018).

In parallel, the increasing use of mobile touchscreen technology, particularly among adults and older adults, has lowered technological barriers (Jenkins et al., 2016). Prior research has shown that individuals with cognitive impairment, including dementia, may be able to interact independently with touchscreen interfaces (Joddrell and Astell, 2016). Tablets, in particular, combine portability, ease of use, and minimal hardware requirements, making them well-suited for brief cognitive assessment even outside traditional clinical settings (Huang et al., 2019).

Many cognitive screening tools place predominant emphasis on memory functioning, a core hallmark of Alzheimer’s disease (Possin et al., 2018). However, beyond memory, domains such as attention, processing speed, executive functioning, and visuospatial abilities are also clinically relevant for early cognitive screening, as deficits in these areas often emerge early across a range of adult neurological conditions, including Parkinson’s disease and vascular cognitive impairment (Possin et al., 2018). Existing digital tools differ markedly in scope, target processes and delivery format. Some tools focuses on specific domains, such as episodic memory (e.g., MemTrax) (Ashford et al., 2019), whereas others provide broad, computer-based cognitive batteries optimized for desktop or laptop administration rather than for rapid touchscreen-based screening (e.g., Automated Neuropsychological Assessment Metrics) (Reeves et al., 2007). In this landscape, TAP-COG was designed as a brief, multidomain, touchscreen-based assessment. Its emphasis on executive–attentional processes (e.g., inhibitory control and resistance to interference), responds to the clinical importance of these functions in disorders where executive dysfunction is an early or prominent feature.

Recent reviews have emphasized the need for home-deliverable digital cognitive screening tools and for the systematic usability evaluation during their development, as usability remains a weak methodological aspects in this field (García-Casal et al., 2017; Robbins et al., 2022). In line with these recommendations, we developed TAP-COG as a brief, tablet-based, web-accessible multidomain screening tool that can be deployed remotely without requiring dedicated software installations or complex platforms. This study aims to (1) present its design and development process and (2) provide preliminary usability data collected in a supervised, in-person setting from a non-clinical adult sample, as a foundational step toward future clinical validation and potential self-administered deployment.

## 2 Materials and methods

### 2.1 Architecture

The TAP-COG battery was developed as a Progressive Web Application (PWA) optimized for tablet-based administration via touchscreen input. Accessible through modern browsers, the platform is compatible with both desktop and mobile devices. The system was built using Flutter and the Dart programming language, both open-source technologies maintained by Google. Flutter enables the creation of cross-platform applications (e.g., Android, iOS, Windows, macOS, Web) from a single codebase, ensuring a consistent and efficient user experience across operating systems. Its architecture supports low-latency rendering and high frame rates, which are essential features for precise stimulus presentation and accurate reaction time measurement in cognitive testing.

The application is organized into two core functional modules: a data management module, responsible for collecting and organizing patients’ demographic information, and a testing module, which presents the cognitive tasks and records responses and results.

Each test within the battery was implemented following the Model-View-Controller (MVC) architectural pattern. Specifically: (i) the *Model* manages graphical and functional test elements; (ii) the *View* renders the user interface; and (iii) the *Controller* handles the logic, determining which elements are displayed and how user input is collected.

To ensure timing precision, a dedicated state management system was implemented. This system tracks changes in the logical state of each task, allowing the controller to trigger specific operations in response to user actions. This approach guarantees timely response handling and accurate reaction time logging.

### 2.2 Patient information management

The application includes a dedicated interface for managing patient demographic data. While the current implementation is available only in Italian, translated screenshots are provided here to illustrate the interface for a broader audience.

**Figure 1:**
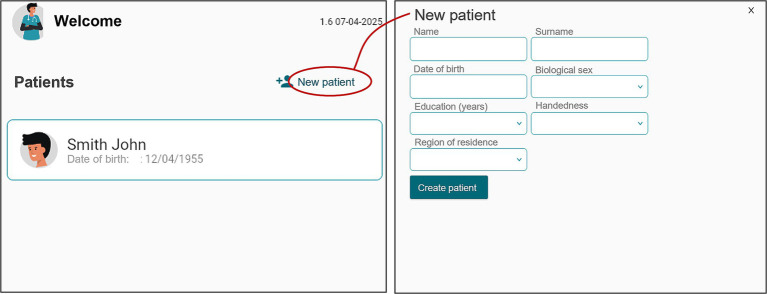
Patient demographic data interface.

Upon first access, users create a new patient profile by selecting ‘New patient’ and entering essential details (e.g., name, dominant hand, region, age, gender, education level), as shown in Figure 1. This information is securely stored and intended to support future score adjustment and personalized interpretation.

**Figure 2:**
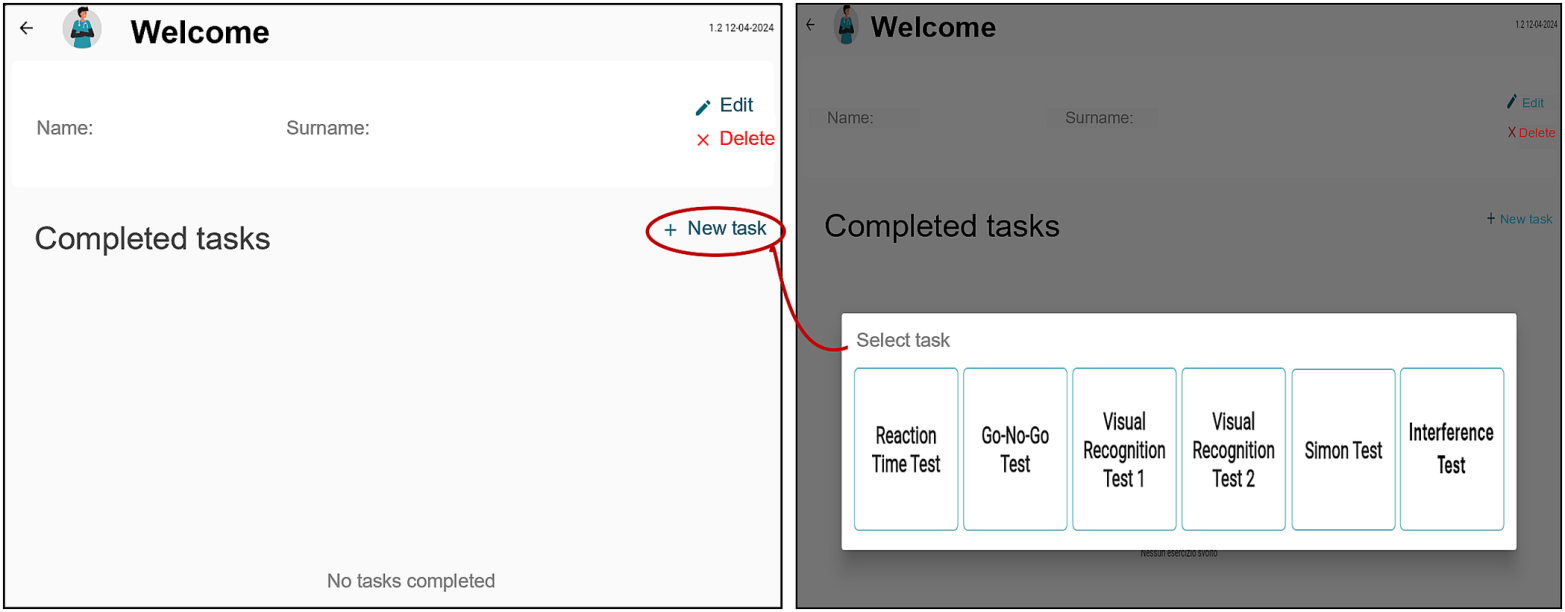
Task assignment interface.

Once a profile has been created, the user can select the “New Exercise” option to assign a task (Figure 2). A panel displaying all available tasks appears, from which a specific task can be selected.

**Figure 3:**
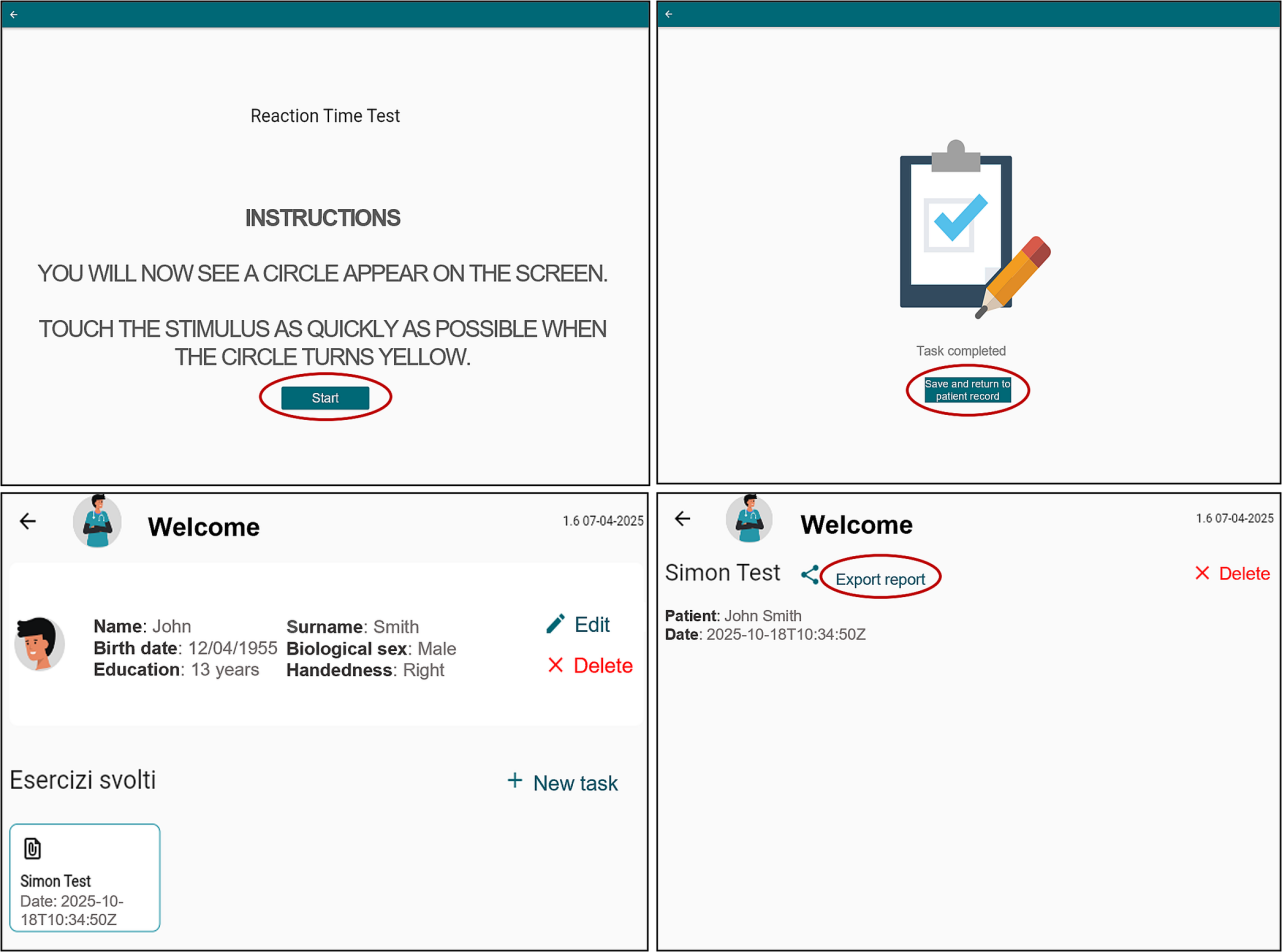
Task execution, completion prompts, and summary display.

The task begins immediately, starting with on-screen instructions followed by the trial phase. After completion, users are prompted to save the results and return to the main page. Each completed task is displayed in the patient profile with a summary of results (Figure 3). Data can also be exported to Excel for further analysis and record-keeping.

### 2.3 Cognitive test

**Figure 4:**
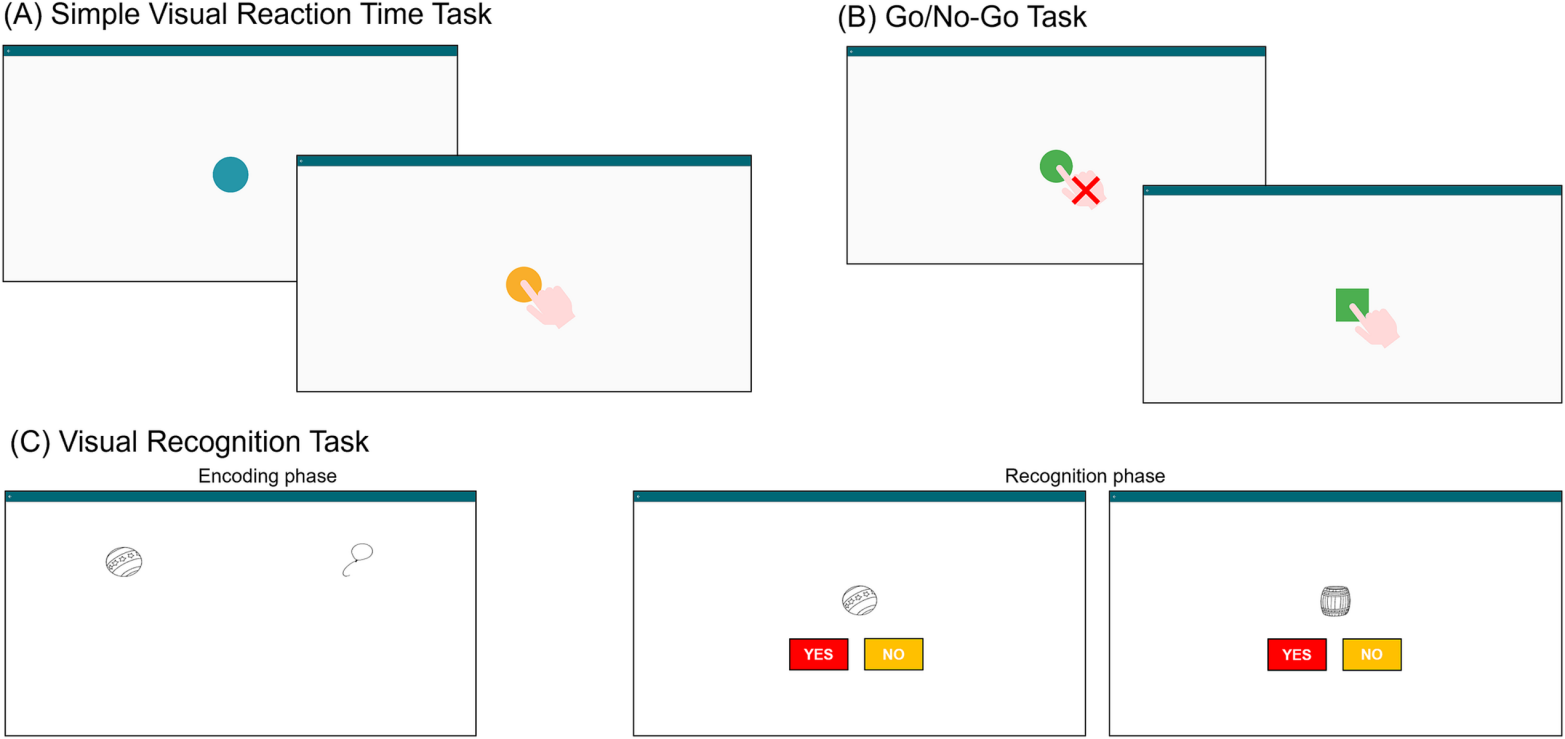
TAP-COG graphical interface: **(A)** Simple visual reaction time, **(B)** go/no-go, and **(C)** visual recognition task (encoding and recognition phases).

**Figure 5:**
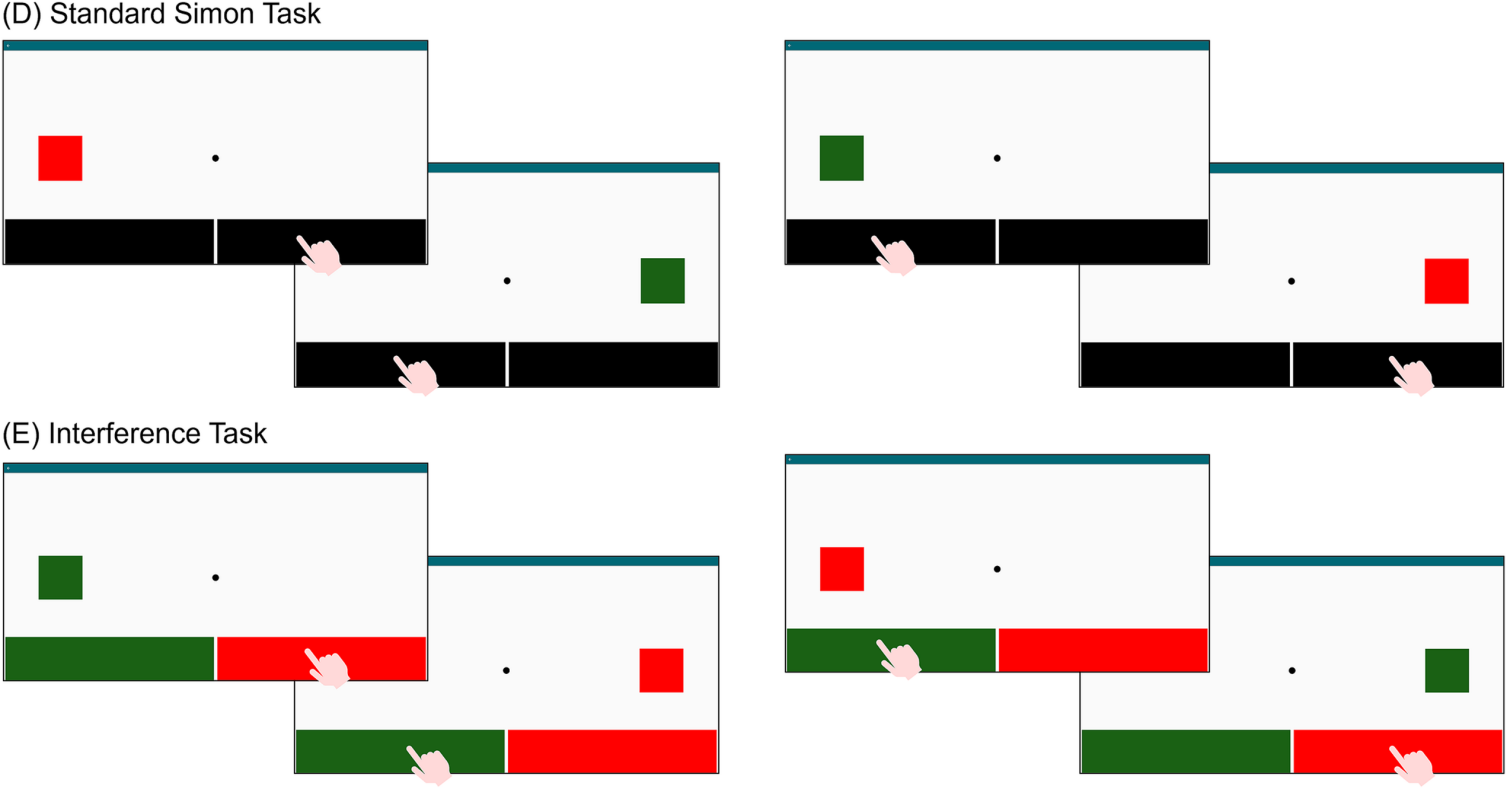
TAP-COG graphical interface: **(D)** Standard Simon task and **(E)** Interference task, including examples of congruent and incongruent trials.

The TAP-COG battery includes five subtests:

- *Simple Visual Reaction Time (sVRT)*: This task assesses processing speed through response latency to visual stimuli. In each of the 35 trials, a blue circle appears on screen and turns yellow after a random interval [4–8 s, according to Barbarotto et al. (1998)]. Participants must tap the circle as soon as it turns yellow (Figure 4A). The system records reaction times and omissions. Faster and more accurate responses indicate better performance. The estimated duration is about 4 min.
- *Go/No-Go*: This task evaluates inhibitory control. Across 70 trials, either a square (target) or a circle (non-target) appears on screen at random intervals (Barbarotto et al., 1998). Participants tap the square while withholding any response to the circle (Figure 4B). Reaction times, and errors (both omissions and false alarms) are recorded. Better performance is reflected in faster and more accurate responses. The duration is approximately 7 min.
- *Visual Recognition (VR)* (Roediger and McDermott, 1995; Ferrucci et al., 2018): This task measures short-term visual memory. It includes eight sequences containing 2 to 8 images, increasing in number and display duration (from 2.5 to 10 s). After viewing each sequence, participants are presented with a set of images that includes a subset of the previous shown stimuli with distractors. The number of images tested increases with the sequence length. Participants indicate for each image whether it was previously presented in the sequence by tapping “yes” or “no” (Figure 4C). Accuracy scores are calculated from correct responses. Two alternate versions are available to prevent learning effects and allow longitudinal tracking. The estimated duration is about 6–8 min.
- *Standard Simon task* (Simon and Wolf, 1963): This task assesses inhibitory control and resistance to spatial interference. On each trial, a red or green square appears on the left or right side of the screen. Participants must tap the left key for green square and the right key for red square, regardless of stimulus position (Figure 5D). Reaction times and errors are recorded, with interference effects – differences between incongruent and congruent trials – considered as indicator of performance. The estimated duration is about 1 min.
- *Interference task*: This task assesses cognitive flexibility and interference control. It consists of three blocks. In the first block, a green key is on the left side of the screen and a red key on the right; participants respond to green and red squares by tapping the corresponding color-coded key (left for green, right for red). In the second block, the mapping is reversed, the red key is now on the left and the green key on the right, and participants respond accordingly (left for red, right for green). In the third block (Figure 5E)—the interference condition—the original layout is restored (green on the left, red on the right), but participants are instructed to respond oppositely: tap left for red and right for green. A total of 30 stimuli is presented. Reaction times and errors are recorded, and interference costs are computed as the difference in performance between incongruent and congruent conditions. The duration is approximately 2 min.

The full battery is self-administered, and total completion time is approximately 30 min.

### 2.4 Preliminary usability testing

An internal testing phase was conducted to verify core system functionality, including proper task presentation, input logging, and especially the accuracy of reaction time recording—crucial for cognitive assessment.

### 2.5 Participants

A preliminary usability study was carried out involving 80 adult participants. Individuals were recruited through convenience sampling from adults accessible to the research team. Inclusion criteria were age ≥18 years and fluent in Italian, while exclusion criteria included severe visual or motor impairments that could prevent tablet use. Ethics committee approval was obtained from the Institutional Review Board (Approval No. 57/25) and informed consent was obtained from all participants involved in the study.

### 2.6 Procedure

Participant completed the full TAP-COG battery individually under the supervision of a neuropsychologist. To assess usability and user satisfaction, the System Usability Scale (SUS) (Brooke, 1996) was administered. This standardized questionnaire provides a quantitative measure of perceived usability, including intuitiveness, ease of use, and overall user experience.

### 2.7 Statistical analysis

Statistical analyses were conducted using IBM SPSS Statistics version 29.0.1.0 (IBM Corp., 2023). Data distribution was examined on raw scores by inspecting skewness and kurtosis values. Skewness values > |1| and kurtosis values > |3| were considered indicative of non-normality (Kim, 2013). As SUS scores did not meet normality assumptions, associations between perceived usability (SUS), cognitive efficiency (MoCA scores), age, and education were examined using Spearman’s rank correlation coefficient (*ρ*). Statistical significance was set at *p* < 0.05.

## 3 Results

We developed a tablet-based cognitive screening designed to assess processing speed, visual attention, inhibitory control, short-term visual memory, and executive functions through a touch-sensitive interface. The comprehensive development process, including architectural design, implementation using Flutter and Dart, and structured programming of cognitive tasks following the MVC framework, resulted in a brief, digital, user-friendly screening tool. The tool is accessible via any browser through a direct link, enabling remote assessment across diverse population. All tasks require non-verbal, manual responses by tapping the touchscreen.

During preliminary testing, we assessed system stability, functionality, and the accuracy of both response timing and data logging. Two issues emerged in the Simon and Interference tasks: if participants inadvertently tapped a response key twice—often due to uncertainty about input registration—the second tap could be erroneously recorded as the response to the subsequent stimulus, even before it appeared. In some cases, this caused stimuli to disappear sequentially, as if responses had already been registered, resulting in inflated error rates and compromised reaction time data. This behavior also produced implausibly fast reaction times (e.g., ~79 ms), reflecting anticipatory recording rather than true responses. To address these problems, we revised the button-disabling logic. Response keys remain inactive until stimulus onset, and any taps occurring prior to stimulus presentation are ignored. Additionally, multiple rapid taps within a trial no longer trigger responses for the current or subsequent stimuli. Follow-up testing confirmed these changes improved task reliability and data integrity.

A total of 80 healthy adult volunteers [mean ± SD, age = 40.75 ± 17.72], all native Italian speakers, participated in the usability evaluation. All participants successfully completed the full TAP-COG battery, which took approximately 20–30 min on average. The sample comprised 58.75% female and 41.25% male. Demographics characteristics and cognitive status are reported in Table 1. After completing the TAP-COG battery, participants filled out the SUS questionnaire. SUS scores indicated good to excellent usability for the majority of participants: 20% (*n* = 16) rated the system ≤67 (marginally acceptable), 20% (*n* = 16) rated it between 68 and 84 (good), and 60% (*n* = 48) rated it ≥85 (excellent).

**Table 1:** Participants’ demographics characteristics and cognitive status.

N	80
Age (years)	40.75 ± 17.72 (21–90)
Sex (M/F)	33/47
Education (years)	15.23 ± 3.2 (5–24)
Handedness (L/R)	6/74
Region of residence (N)	
Northern Italy	71
Central Italy	3
Southern Italy	6
MoCA	28.45 ± 1.72 (19–30)

Scores are reported as mean ± standard deviation. N, number of participants; M, male; F, female; L, Left; R, Right MoCA, Montreal Cognitive Assessment.

To explore potential associations between usability and participant characteristics, we conducted Spearman’s Rho correlations (see Table 2). No significant associations were found between SUS scores and age (*ρ* = −0.10, 95% CI [−0.31, 0.12], *p* = 0.367), years of education (*ρ* = −0.09, 95% CI [−0.30, 0.13], *p* = 0.440), or MoCA (*ρ* = 0.05, 95% CI [−0.17, 0.27], *p* = 0.654).

**Table 2:** Correlations between age, education, cognitive efficiency, and system usability scale scores.

Variable	Spearman’s ρ / *p*-value	SUS
Age	*r_s_*	−0.10
	*p*	0.367
Education	*r_s_*	−0.09
	*p*	0.440
MoCA	*r_s_*	0.05
	*p*	0.654

*r_s_*, Spearman’s Rho; *p*, *p*-value; SUS, System usability scale; MoCA, Montreal cognitive assessment.

## 4 Discussion

The present work aimed to introduce the TAP-COG battery, a tablet-based tool designed to assess cognitive functioning through a set of five digital tasks. Its development was driven by increasing body of evidence supporting the advantages of digital neuropsychological assessment over traditional paper-and-pencil methods. These benefits include standardized administration, automated and objective scoring, precise stimulus presentation and response tracking, reduced costs, and enhanced ecological validity (Woo, 2008; Parsons and Parsons, 2016). Additionally, digital tools offer new opportunities for the early detection of cognitive impairment (Wang et al., 2021), which is crucial for patients and caregivers to facilitate future planning, implement targeted interventions, and mitigate modifiable risk factors that may accelerate cognitive decline (Huntley et al., 2015).

The TAP-COG battery evaluates processing speed, inhibitory control, and short-term visual memory using five tasks: a simple reaction time task, a go/no-go task, a visual recognition task, a Simon task, and an interference task. Developed as a PWA using Flatter, the system ensures cross-platform compatibility, high responsiveness, and precise temporal control, which are essential features for reliable cognitive assessment. The battery delivers a standardized, non-verbal, touchscreen-based experience, making it particularly suitable for minimally supervised or remote use. Importantly, TAP-COG relies exclusively on visual stimuli and manual (touchscreen) responses, minimizing the influence of confounding variable such as language proficiency or hearing ability, factors that may disproportionately affect older adults or clinical populations (Tagliabue et al., 2023). This design also facilitates remote administration by eliminating the need for additional hardware (e.g., speakers, audio) and multimodal synchronization of stimuli.

A preliminary usability study involving 80 healthy adults demonstrated promising results. SUS scores indicated that 80% of participants rated the platform as either good or excellent in term of interaction with the digital system, while the remaining considered it as problematic or minimally acceptable. Notably, usability ratings were not significantly associated with participants’ age, education level, or general cognitive functioning, suggesting that the tool may be broadly accessible, possibly also to individuals with varying levels of technological familiarity or cognitive functioning. Touchscreen interfaces reduce reliance on external input devices such as keyboards or mice, minimizing visuomotor coordination demands (Joddrell and Astell, 2016) and promoting more natural, user-centered interaction (Canini et al., 2014). This may reduce test-related anxiety or frustration, thereby enhancing compliance and data quality (Wild et al., 2008; Bauer et al., 2012). These findings align with prior research on tablet-based cognitive assessment, which has shown that such platforms are generally perceived as intuitive, engaging, and accessible by individuals with limited digital literacy or cognitive decline (Canini et al., 2014; Lussier et al., 2021; Massetti et al., 2023).

However, an important limitation must be acknowledged. The present sample was characterized by relatively high cognitive efficiency and educational attainment, as well as relatively young average age. As such, the usability performance observed here may not fully extrapolate to the intended target population of older adults or individuals with cognitive impairment. Age-related visuomotor slowing, reduced attentional control, and lower familiarity with digital devices can substantially increase the likelihood of usability difficulties in older users, and these challenges may be further magnified in those with mild cognitive impairment or dementia. Recent findings indicate that touchscreen-based cognitive tests can still be feasible in these groups (Joddrell and Astell, 2016), but technical barriers, such as slower comprehension of instructions, increased need for repetition, or difficulty maintaining task engagement, occur more frequently and can meaningfully affect the quality of performance and data validity.

A current limitation of TAP-COG is the absence of mandatory practice trials and the use of a fixed task order, and the lack of structured qualitative feedback on participants’ experience. Incorporating practice trials and break screens between task blocks could help participants internalize instructions, reduce cognitive load, maintain engagement. Additionally, the inclusion of more detailed qualitative evaluation could better capture usability and potential fatigue effects, particularly in later tasks and in users with reduced processing speed or attentional control. Overall, these results provide a solid foundation for the continued development of TAP-COG. Future research will focus on evaluating the psychometric properties of TAP-COG, including construct and concurrent validity, reliability, and the establishment of normative data. Initial validation will be extended to older healthy adults, followed by clinical cohorts with neurodegenerative or psychiatric conditions. To assess construct validity, TAP-COG performance will be compared with established paper-and-pencil neuropsychological tests targeting the same cognitive functions. Comparing performance across formats will help determine the extent to which TAP-COG accurately captures the intended constructs. Future studies should also evaluate whether the tool performs equivalently when administered remotely versus in-person, to ensure its reliability across different administration contexts. Furthermore, to ensure broader generalizability and to better capture the usability experience of the intended population, future studies should include older adults and individuals with lower educational backgrounds. These efforts will support the development of a robust, accessible, and scalable tool for both clinical and research application.

## 5 Conclusion

This study introduced TAP-COG, a brief, tablet-based cognitive tool designed for easy, remote use. Initial results show good usability among healthy adults, though the sample was relatively young and well-educated. Future research will focus on psychometric validation and expanding the battery to cover a broader range of cognitive domains, aiming to establish TAP-COG as a reliable and accessible resource for both research and clinical applications. Subsequent studies will also evaluate its feasibility and usability in ecological or ambulatory settings, as well as in clinical populations, to support real-world deployment and further validate the tool.

## Data Availability

The datasets presented in this study can be found in online repositories. The names of the repository/repositories and accession number(s) can be found at: University of Milan Dataverse repository, https://doi.org/10.13130/RD_UNIMI/WIUD6W.
